# User perspectives on a psychosocial blended support program for partners of patients with amyotrophic lateral sclerosis and progressive muscular atrophy: a qualitative study

**DOI:** 10.1186/s40359-019-0308-x

**Published:** 2019-06-15

**Authors:** Jessica de Wit, Sigrid C. J. M. Vervoort, Eefke van Eerden, Leonard H. van den Berg, Johanna M. A. Visser-Meily, Anita Beelen, Carin D. Schröder

**Affiliations:** 1 Center of Excellence in Rehabilitation Medicine, Brain Center Rudolf Magnus, University Medical Center Utrecht, Utrecht University and De Hoogstraat Rehabilitation, Utrecht, The Netherlands; 2 Department of Imaging and Oncology, University Medical Center Utrecht, Utrecht, The Netherlands; 3 Department of Neurology, Brain Center Rudolf Magnus, University Medical Center Utrecht, Utrecht, The Netherlands; 4 Department of Rehabilitation, Physical Therapy Science & Sports, Brain Center Rudolf Magnus, University Medical Center Utrecht, Utrecht University, Heidelberglaan 100, 3584 CX Utrecht, The Netherlands

**Keywords:** Caregivers, Amyotrophic lateral sclerosis (ALS), Progressive muscular atrophy (PMA), Acceptance and commitment therapy, Psychological distress, Qualitative research

## Abstract

**Background:**

Partners are often the main caregivers in the care for patients with amyotrophic lateral sclerosis (ALS) and progressive muscular atrophy (PMA). Providing care during the progressive and fatal disease course of these patients is challenging and many caregivers experience feelings of distress. A blended psychosocial support program based on Acceptance and Commitment Therapy was developed to support partners of patients with ALS and PMA. The aim of this qualitative study is to gather insight into experiences with different components of the support program (program evaluation) and to discover what caregivers gained from following the program (mechanisms of impact).

**Methods:**

Individual in-depth interviews, about caregivers’ experiences with the support program were conducted with 23 caregivers of ALS/PMA patients enrolled in a randomized controlled trial designed to measure the effectiveness of the blended psychosocial support program. The program, performed under the guidance of a psychologist, consists of psychoeducation, psychological and mindfulness exercises, practical tips and information, and options for peer contact. Interviews were audio-recorded, transcribed verbatim and analyzed thematically.

**Results:**

The program evaluation showed that caregivers perceived each component of the program as beneficial but ambivalent reactions were expressed about the mindfulness exercises and peer contact functions. Caregivers expressed the need for a more personalized program with respect to the order and timing of the modules and wanted to continue the support program for a longer time. The main mechanism of impact of the program that caregivers reported was that they became more aware of their own situation. They further indicated that the program helped them to perceive control over the caregiving situation, to accept negative emotions and thoughts, to be there for their partner and feel acknowledged.

**Conclusions:**

The blended psychosocial support program for caregivers of patients with ALS/PMA is valued by caregivers for enhancing self-reflection on their challenging situation which stimulated them to make choices in line with their own needs and increased their feeling of control over caregiving. The different components of the program were overall appreciated by caregivers, but the mindfulness and peer support components should be further adapted to the needs of the caregivers.

**Trial registration:**

Dutch Trialregister NTR5734, registered 28 March 2016.

**Electronic supplementary material:**

The online version of this article (10.1186/s40359-019-0308-x) contains supplementary material, which is available to authorized users.

## Background

Caregivers of patients with amyotrophic lateral sclerosis (ALS) or progressive muscular atrophy (PMA) are confronted by many challenges during the progressive and fatal disease course of the patient. They are faced with physical deterioration and possible cognitive and behavioral changes in patients, which results in increasing demands on the caregiver [1]. Caregivers who experience increasing demand but do not feel in control over the caregiving situation are more likely to experience psychological distress according to the demand and control model [2, 3]. Research shows that caregivers experience high levels of distress and caregiver burden [4, 5].

Although caregivers express a need for psychosocial support [6] supportive evidence based interventions for these caregivers are lacking [7]. A psychosocial support program was developed to diminish feelings of distress in caregivers of patients with ALS and PMA by enhancing caregivers’ feelings of control over the caregiving situation [8]. The support program is based on Acceptance and Commitment Therapy (ACT) [9]. ACT encourages individuals to accept unwanted private events which are out of personal control and to identify important values in life in order to pursue these values which might help caregivers of patients with ALS or PMA [9]. The program consists of a combination of face-to-face-, online- and telephonic contact (i.e. blended support). The content of the support program is originated from an existing intervention for partners of people with cancer [10] and adapted to the needs of caregivers of patients with ALS and PMA. The effectiveness of the support program is currently being evaluated in a randomized controlled trial (RCT) in which caregiver-patient dyads are included. In order to understand the mechanisms of the impact of the intervention, a qualitative evaluation study regarding the experiences of caregivers with the support program, alongside the trial, is important [11–13]. Furthermore, insight into caregivers’ experiences with the specific components of the intervention is valuable for implementation of care for these caregivers in the future [13]. Therefore, this study explores caregivers’ experiences with a blended psychosocial support program for caregivers of patients with ALS/PMA. We aimed to gather insight into experiences with the different components of the program (program evaluation) and to discover what caregivers gained from following the support program (mechanisms of impact).

## Methods

### Study design

This qualitative study is embedded in an ongoing RCT investigating the effectiveness of the support program on psychological distress of caregivers (NTR5734). The protocol of the RCT is described in detail elsewhere [8]. The trial includes 148 caregivers and 101 patients.

### The support program

The support program consists of an introductory face-to-face appointment with a psychologist, six psychologist-guided online modules and a concluding telephone contact with the psychologist (the content of the program is represented in Additional file 1). The face-to-face session was held at the residence of the caregiver. In this session, the psychologist explained the purpose of the intervention, received information about the caregiver’s situation, demonstrated the online program and established a working relationship with the participant. After this session, the participant started with the first of 6 online modules. Every module was focused on a specific topic and consisted of different components (see Table 1). After finishing a module, caregivers received feedback from the psychologist. The program ended with a telephone call in which the caregiver had the opportunity to ask advice or discuss their remaining questions. The program was scheduled to be completed within 8 weeks. However, if caregivers needed more time, this could be extended to 12 weeks. The support was provided by three psychologists who were trained to provide the intervention and who were not related to the multidisciplinary ALS care teams.

**Table 1:** Content of each online module

Components	
• *Psycho-education and exercises* Information directed at the theme of the module with psychological exercises based on ACT	
• *Mindfulness exercises* Listening exercises to train conscious awareness and attention from one moment to the next moment	
• *Practical information, tips and references* A list of relevant websites, organizations and other sources of information and support associated with the theme of the module	
• *Contact with peers* 1. Sending private messages using a personal profile 2. Sharing tips and advice with regard to the topic of the module with fellow participants via a forum	
• *Feedback of the psychologist* After finishing a module, the participant receives feedback on the completed exercises, a reflection on the progress and a reaction to any questions or difficulties via a text message	

Abbreviation: *ACT* Acceptance and Commitment Therapy

### Sample and recruitment

Participants were purposively sampled from the 67 eligible caregivers in the RCT. Caregivers in the RCT met the following criteria: 1) the caregiver is the partner of the ALS or PMA patient; 2) the caregiver is 18 years or older; 3) the caregiver is proficient in Dutch to fill out the questionnaires; 4) the caregiver has internet access; 5) the caregiver has consent of the patient to participate, as the caregiver answers questions about the wellbeing of the patient. Caregivers in the RCT who completed or dropped out of the support program and who finished the third measurement (approximately 6 months after baseline), were selected. Only caregivers who completed the third measurement of the RCT were invited, as we did not want to influence the trial assessing the effectiveness of the intervention. Maximum variation in the sample was obtained by selecting caregivers with a wide distribution range with regard to age, gender and the physical and behavioral impairments of their partner. Selected caregivers were asked to participate via e-mail. In case of refusal, another caregiver was purposively sampled from the database as a replacement. In total, 40 caregivers were invited for an interview and 23 (57.5%) agreed to participate. Twelve caregivers did not respond to the invitation and five refused to participate. Reasons for refusal were: not willing to spend time on the study (1), afraid it would be too emotional (1), and having a partner in a critical phase of the disease (1). Two caregivers did not report a reason.

### Data collection

Semi-structured interviews were conducted by telephone using an interview guideline. The interview guideline was developed with open questions related to caregivers’ experiences with the support program in general, and the specific components of the program (Additional file 2). The interview guideline was peer reviewed by the research team and further refined during the iterative process and based on emerging themes. Participants who dropped out of the support program where interviewed about their reasons for dropping out and their experiences with the program.

Caregivers who were interested in participating received the interview questions per e-mail as well as a summary of the content of the support program to help retrieve their memory of the intervention and to enhance reflection.

Interviews were conducted between June and September 2018 by a master student Health and Life Sciences who had been trained in conducting interviews (EE). The interviewer was not known to the participants prior to the start of the interview. All interviews were audiotaped. The duration of the interviews with caregivers who completed the support program ranged from 38 to 82 min (*m* = 57 min). Interviews with participants who had dropped out were shorter, ranging between 17 to 26 min in length (*m* = 22 min). During and directly after the interviews, memos were made to capture ideas about emerging themes and refinement of the interview guideline. Interviews were held until saturation was reached and confirmed during analysis of the last three interviews [14].

Demographic characteristics of the caregivers and disease-related characteristics of the patients were gathered in the context of the RCT. Insight into the severity of the patient’s disabilities was collected via the Amyotrophic Lateral Sclerosis Functional Rating Scale-Revised (ALSFRS-R) [15]. In this validated questionnaire, higher scores denote better physical functioning. Behavioral changes in ALS or PMA patients were assessed using the Amyotrophic Lateral Sclerosis-Frontotemporal Dementia-Questionnaire (ALS-FTD-Q) [16]. The validated questionnaire asks the caregiver to compare the patient’s current behavior with his/her behavior 3 years ago and higher scores indicate more behavioral changes. Both questionnaires were completed by caregivers.

### Data analysis

Interviews were analyzed thematically according to the six phases described by Braun and Clark [17]. The analytic steps and the roles of the authors in this process are presented in Table 2. The data regarding the program evaluation were analyzed according to the first two steps. No in-depth thematic analyses were conducted on this data as we wanted to provide a description of the experiences with the different components. The software program NVIVO 10 was used to support data analysis [44].

**Table 2:** Phases of thematic analysis according to Braun and Clarke

Phase	Description of process and role of authors
1. Familiarizing with the data	Interviews were transcribed verbatim and the accuracy of transcripts was checked by comparing the audio recordings with the transcripts (JW, EE). Transcripts were read and re-read by three authors (JW, EE, SV) and memos of initial ideas about themes and refinement of the interview guide were made and discussed (JW, EE, SV, CS). The authors had different professional backgrounds; i.a. psychology, nursing science and health sciences.
2. Generating initial codes	Transcripts were broken down into fragments based on content, and these fragments were labelled with codes by researchers independently (JW, EE). After every three interviews, results of their coding were compared and discrepancies discussed leading to consensus. A third researcher, who is an expert in qualitative research (SV), coded seven interviews. Results of the codes were discussed during meetings in which the researchers worked towards consensus about the coding and interpretations of the data (JW, EE, SV, CS).This approach established researchers’ triangulation and increased the depth and credibility of the analysis.
3. Searching for themes	Codes were collated into potential themes whose relevance emerged across the interviews (JW, EE). A potential description of the main and subthemes was made. Potential themes were discussed in joint meetings (JW, EE, SV, CS).
4. Reviewing themes	Potential themes were reviewed for consistency with the codes and entire data to ensure they reflected the entire dataset (JW, EE). Inconsistencies were discussed and potential themes were further refined (JW, EE, SV, CS).
5. Defining and naming themes	The specific content of each theme was further worked out using the transcripts, and themes were named and defined (JW, EE, SV, CS).
6. Producing the report	Two researchers (JW, EE) wrote a first draft of the scientific report and selected quotes to illustrate themes. Two authors reviewed the report (CS, SV) and adjustments were made. This process was repeated until consensus was reached. The report was sent to the other members of the research team (AB, LB, JV) for critical assessment, and their feedback was processed.

The 15-point checklist of Braun and Clarke was used to confirm the correct application of the six phases of thematic analysis (see Additional file 3) [17]. Reporting in this paper is in accordance with the Standards for Reporting Qualitative Research (SRQR) checklist (O’Brien et al. [18]) (see Additional file 4).

## Results

The majority of participants were women (65%). The age of the caregivers ranged between 33 and 80 years (overall mean 59.6 years). Most patients were diagnosed with ALS (70%). The personal characteristics of the caregivers and their patients are listed in Table 3.

**Table 3:** Characteristics of interviewed partners

	Completers (*n* = 17)	Drop outs (*n* = 6)
Gender, *n* (%)		
Female	12 (70.6)	3 (50.0)
Male	5 (29.4)	3 (50.0)
Age in years, mean (SD)	59.9 (10.9)	58.7 (13.4)
Education level, *n* (%)		
Low	1 (6.0)	–
Medium	8 (47.0)	3 (50.0)
High	8 (47.0)	3 (50.0)
Diagnosis partner, *n* (%)		
ALS	13 (76.5)	3 (50.0)
PMA	4 (23.5)	3 (50.0)
Time since diagnosis in months, median (range)	33 (9–253)	35 (12–82)
Parameters patient		
ALSFRS-R, median (range)	25 (4–44)	24 (5–34)
ALS-FTD-Q, median (range)	11 (0–38)	15.5 (8–33)

Educational level: low = did not complete secondary school-completed low level secondary school; medium = completed medium level secondary school; high = completed upper level secondary school and/or university degree

Abbreviations: ALS-FRS-R, Amyotrophic Lateral Sclerosis Functional Rating Scale-Revised; ALS-FTD-Q, Amyotrophic Lateral Sclerosis-Fronto Temporal Dementia-Questionnaire

**Fig. 1:**
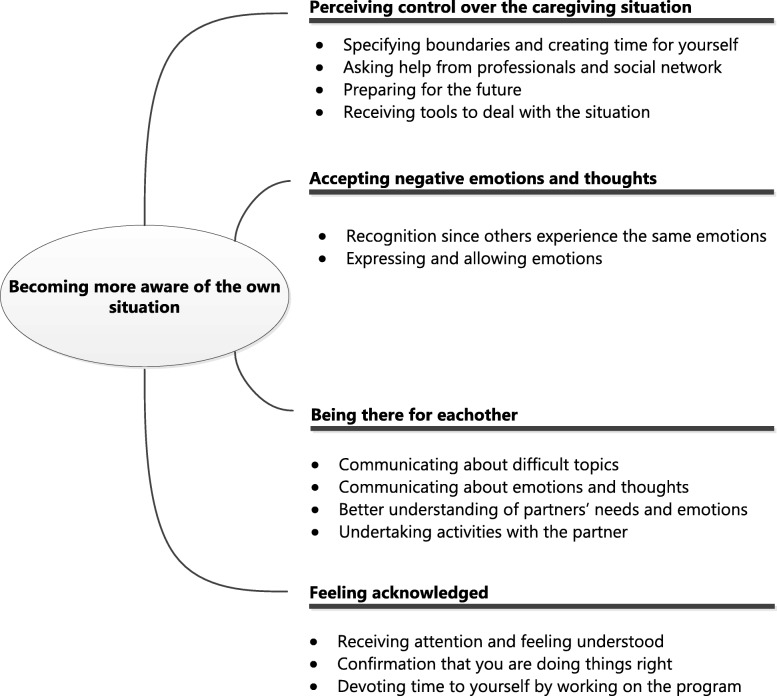
Overview themes and subthemes Mechanisms of impact

The results are presented in two main sections. The first section contains the program evaluation: user experiences with the different components of the program (Table 1). Three important topics were added to provide a complete overview: “receiving online support”, “timing of the intervention” and “flexibility and length of the program”. The second section presents the themes regarding the mechanisms of impact (i.e. what caregivers gained from following the program) (see Fig. 1).

### Program evaluation

In general, caregivers felt the program contained all the crucial themes relevant to their situation. Caregivers said there was coherence between the different modules, each module offering more in-depth information. The fact that the program contained disease-tailored themes and information was appreciated by caregivers:

“*I think it is very good that something like this exists, because my husband has the disease, but as a partner you will be dragged along with it. […] So I feel it’s good that there is attention for that, and that you can receive some support for it. And yes of course it [the program] was very much related to the ALS disease. So it also deals very specifically with the course of that disease. So that really did help me.*” (*Respondent (R)5)*

### Receiving support online

Most of the caregivers found the program easy to use and some mentioned that it had a user-friendly interface. The fact that each module had the same structure, and that each segment mentioned the time it would approximately take to complete it, was valued by caregivers.

There were some technical issues caregivers encountered, such as problems with saving the answers to the exercises or difficulties in leaving comments or tips for peers. These issues made some caregivers feel that the program was difficult to use. Some of them said they found computers in general difficult to use and were, therefore, struggling with the program:

*“I encountered my lack of experience with these sorts of things [computers]. […] It’s just that I don’t know how to work with that.” (R11)*

By receiving the program online, it provided caregivers flexibility in pacing themselves, in contrast to traditional face-to-face support. The majority of caregivers appreciated the fact that they were able to work on the program whenever and wherever they wanted. They were also able to pause and continue with the program at any moment. Some valued this opportunity to think about the exercises and reflect upon their answers before submitting them. Being able to receive support at home was an important benefit of the program according to the caregivers. Some of them mentioned they would not have been able to receive support in a traditional care setting, such as via the specialized ALS team, because of their care responsibilities and the inability to leave their partner at home alone:

*“I came to a point where I thought: I need something. Something that gives me air and makes me think. But then there’s the problem you often face: I can’t leave. […] The fact that it’s online, it’s a small step. It’s different from conversations with someone, so it saves a lot of time. Online means you can do it at the moments that work for you.” (R14)*

Some caregivers would have preferred face-to-face support instead of online support. According to them, interaction via de computer lacks spontaneity and written answers come across differently due to the lack of verbal and non-verbal communication.

### Timing of the intervention

The majority of caregivers perceived the timing of the intervention as appropriate and found the topics relevant in their situation:

*“I think the program came at a good time. That everything is still relatively new for you, and can put your own things into perspective and that you receive support. Otherwise, you will keep going in circles for too long.”(R17)*

However, some caregivers of partners in a more advanced stage of the disease would have preferred to have received the intervention earlier. Receiving the intervention too late in the disease course was the main reason mentioned for dropping out of the program. A few felt the program did not offer them new information now, while it could have prepared them for what was to come had it been provided earlier. Others were so taken up by care tasks that they had limited time left for other activities. With little time available, some caregivers said the program started to feel burdensome:

*“Usually I would do it [the program] almost at night, at the end of all the chores I have. And then I would start working on it, but it was just an extra chore added to my list. So I couldn’t manage and I started falling behind etcetera and it started to become more of a burden than an aid.” (Drop-out Respondent (DR)2)*

Most caregivers of partners in the early disease stage found the intervention to be helpful as it pointed out what could happen in the future. However, some found that the intervention was not helpful to them yet as the need for support had not yet arisen and they preferred to spend time on other activities instead of focusing on the disease.

The subject, ‘end of life’, which is discussed in the last module, was difficult for many caregivers to read or discuss with their partner. Some caregivers did not want to follow the last module at all, skipped the exercises, or saved it to do it at a later moment when it would be more relevant.

### Flexibility and length of the program

Caregivers appreciated the fact that exercises were not mandatory; they could freely choose which exercises they wanted to complete. This gave them the freedom to focus on elements that were relevant to their situation and to skip elements that were considered to be too confronting, not applicable, or not yet relevant. Some caregivers indicated that they would have preferred to decide for themselves when they followed which modules, instead of following the modules in a fixed order. This would enable caregivers to select the modules most relevant to them at that moment. While the majority of caregivers felt there was sufficient time to complete the modules, some reported they would have liked to have more time in between modules to reflect upon the information received and to reduce the feeling of time pressure that some experienced.

Although most caregivers thought the length of the intervention was sufficient, caregivers expressed the desire to continue the intervention for a longer period of time or to have more follow-up appointments after the end of the program. Caregivers felt this would help them to retain the information longer and enhance the long-term effect of the program:

*“Those 6 weeks, they really helped to sort things out again. But you gradually notice that you start to forget things. […] Things change so much with ALS. When I look at how I experienced it in the beginning and in the final phase, it is so different. So I would like to give it as advice to offer the program several times. It simply helps you to make conscious choices.” (R14)*

### Psychoeducation and psychological exercises

Caregivers found the psycho-educational information in each module useful, as it offered a clear introduction to the theme of the module and it provided them with sufficient information to complete the exercises. Through the exercises, caregivers felt they received the tools to cope with difficult situations, emotions and negative thinking. Some caregivers indicated they had re-examined the information and exercises later on when they encountered issues related to these themes in their daily life.

*“Sometimes I read or heard something and thought: ‘I’ve had this in the modules, let’s take a look’. And then I went back and looked at it [the psycho-educational information] and I found something there. So it was information I re-examined afterwards. I think that is positive, that you can look up information about situations you encounter” (R10)*

### Mindfulness exercises

The experiences with the mindfulness exercises were mixed. The majority of caregivers did not perceive the exercises as beneficial, and some caregivers said these led to adverse effects, including stress or feelings of restlessness. Finding the time to complete the mindfulness exercises was difficult for some caregivers. For others, the voice of the narrator was unpleasant or they found the text, that was read out, too woolly:

*“I found the mindfulness, well, it just is not for me. That man that is speaking so unctuously, it made my hairs stand on end.” (R7)*

However, there were caregivers who did appreciate the mindfulness exercises. For them it was a valuable part of the program that provided them with a feeling of relaxation and calmness.

### Practical information, tips and references

This component provided many caregivers with new links to relevant websites and information. One caregiver, for example, said this information helped her finally sort out an application for a personal care budget request. Caregivers felt that it provided a clear overview with useful and reliable information, as it had been developed by professionals in ALS:

*“I found the information interesting and I’ve read it all. Before that, I thought, I’m not going on the Internet anymore. But then you think, this comes from the ALS Center, from professionals, I can read that. I have more faith in that, rather than the vague stories that you see on the Internet.” (R12)*

By saving this information on their computers, caregivers said, they were able to use the information provided later when needed. Other caregivers said they skipped this section because they did not have time to read it, or they felt it was too much to read at the end of a module.

### Contact with peers

Caregivers could have contact with other caregivers in two ways: sending each other private messages or leaving tips and advice at a forum for others to read and react to. Caregivers who contacted others via direct messaging said this contact was valuable as it provided an opportunity to share their story and experiences. This one-on-one contact provided caregivers with recognition and acknowledgement: the notion that others were in the same situation, and that they had the right to feel the way they did:

*“I have read pieces of text from other caregivers with tears in my eyes. Not because they are in a terrible situation but tears of joy of recognition. I can say straight away that that is the most important thing, because there you do see the recognition. In the outside world everyone goes on with their own lives, there you cannot find this recognition and acknowledgement.” (R13)*

However, most caregivers said that they did not feel the need to get in touch with peers through the program. For many, the threshold to contact someone via private messaging was too high, as they felt insufficiently informed about the situation of their fellow users. Some caregivers with a partner in an early disease stage felt no questions had yet arisen or felt it would be too confronting to talk to a peer caring for an ALS patient in a more advanced stage.

The tips and advice left by others were often considered to be too generic and therefore not useful. According to some caregivers, giving or receiving advice is not useful since each individual situation is different:

“*There were tips from people in a much more advanced stage of the disease. […] That did not add anything for me. It is often about the life of someone else, I am not that interested in it and it doesn’t offer me anything.” (R12)*

Others strongly valued the option to leave tips and advice for others as it gave them the opportunity to share experiences with others in a similar situation:

*“Every week I left tips for others and I always read the tips from other participants. I found that one of the most fun parts of the program. I also considered that as very important. The reason for that may be that I did not encounter ALS in my personal environment, while I did feel the need to share experiences.” (R14)*

### Contact with the counsellor and feedback

The majority of caregivers said they appreciated the home visit of the counsellor at the beginning of the support program and saw the home visit as an essential part of the support program. First, it provided caregivers with a face to go with the name of the counsellor. Second, the brief instructions given on how to use the program helped caregivers to get started. Third, caregivers felt that by seeing their situation, the counsellor was provided with context to the answers caregivers would send in and receive feedback on. As some caregivers indicated, this face-to-face meeting created a relationship of trust and understanding:

*“It’s nice to know who will be reading your things, and you would be more open than if you weren’t to know who’s behind it. I would have been more closed if I had not known who was on the other side.” (R17)*

Caregivers felt the contact with the counsellor was pleasant, as there was a short line of communication and caregivers felt comfortable asking the counsellor questions if necessary. Some caregivers mentioned that having a counsellor with knowledge on ALS was important, as they felt this provided the counsellor with a better understanding about the problems they might encounter as caregivers.

Many caregivers found the feedback on the exercises provided by the counsellor valuable because it helped them to reflect on their situation and offered them advice. The feedback confirmed the validity of their feelings and actions, which made them feel understood and encouraged. The majority of completers said they considered the feedback as a crucial part of the intervention, as it gave them insight into their own thoughts and feelings and it motivated caregivers to continue with the intervention. An important aspect of the feedback was that it applied to the caregivers’ personal situation, and included elements of what they had filled in during the exercise. This made caregivers feel like they were listened to and that they were being taken seriously:

*“She provided feedback and tips, and all in a very pleasant manner, like, ‘oh think about this’ […] It made me feel like a lot of care was put into it. That she really looked at it seriously.” (R1)*

However, others felt that the feedback was too brief and superficial. Their main objection was that the feedback was too recapitulatory: it summarised and repeated their answer back to them rather than providing them with new insights. As one caregiver illustrates:

*“I think I wanted or hoped for more in-depth feedback. […] regarding the quality of the feedback I’d sometimes think hmmm.” (R13)*

### Mechanisms of impact

#### Becoming more aware of the own situation

Caregivers became more aware of their own situation and reflected upon their situation through the support program. Due to the hectic and demanding care situation, caregivers were in a flow of providing care and as a result little attention was paid to self-reflection. The program encouraged caregivers to stand still and reflect, which they appreciated. The exercises required them to think about and describe their own situation in concrete terms. This forced caregivers to actively evaluate their current situation and to verbalize what they would like to see or do differently.

*“I perceived the program as very useful because it made me think about what I want to do. I had to face the facts; how is it going right now? Are there things that I would like to do differently? That helped me.” (R14)*

In addition, the program offered new insights and perspectives, through the information provided and through the tips of other caregivers.

*“I also got a bit of an idea of how other caregivers were looking at the care situation and what kind of other perspectives there are. I liked that. [...] I realized, yes, it is also possible to see it in another perspective.” (R9)*

#### Perceiving control over the caregiving situation

Due to the program, caregivers reflected on their caregiving role now and in the future, the tasks they performed and the division between one’s own time and caregiving. The program taught caregivers to recognise and set their personal boundaries. By indicating their boundaries, caregivers said they learned to keep control of their own life. One caregiver gave the example of cancelling work appointments because they were too demanding in terms of time and energy. The program helped caregivers to consciously think about the choices they were making and thereby define what was important to them. As another caregiver illustrates:

*“What is really important? Well, you have to learn to discover that yourself and this (the program) helped with that. Like, you can also just say ‘no’. Or you can tell your friends: ‘you can’t come to my house right now, I’ll come to you’.” (R3)*

The program also made caregivers aware that asking others for help could alleviate the demands that were placed on them and created more time for themselves. Asking for help from caregivers of professionals helped them to regain control over their situation.

*“We do things more consciously and now also call in help from friends, family and neighbours. People offer help and ask whether they can do something for us. In the beginning we kept that off, but now we also ask people for help ourselves.” (R12)*

Caregivers felt that the assignments and themes covered in the program provided them with information to prepare for the future and provided them with tools to deal with future situations. Thereby, it gave caregivers more confidence in being able to handle the future and helped them accept the difficulty of what lies ahead:

*“And if it comes, then you’ll think: ‘I’ve read this’, and it won’t come as a surprise. I think that is much better, you have to be well prepared. And then it’s easier to carry.” (R10)*

#### Accepting negative emotions and thoughts

Reflecting upon their situation provided caregivers insight into their thoughts and emotions. The intervention helped caregivers realize they were not alone in experiencing negative emotions and thoughts, and that these were valid to have in their difficult circumstances. This helped them to deal with these emotions and thoughts and caregivers felt they were able to accept this:

*“[…] that you are not alone, that the feeling you have is right. Fears and other emotions that you have, that they are right and not different from others. Just the confirmation of this, and the description of the emotions and information in the modules. At the moment it made me more calm and I think that I am generally calmer now in respect to the disease.” (R17)*

Reading that these emotions and thoughts were normal in these circumstances also lowered the threshold for caregivers to express these emotions and thoughts.

*“It became clear to me that I do not have to suppress my emotions. [...] One of the things that has been confirmed was that you cannot help it [having these emotions] so you don’t have to push them away.” (DR3)*

#### Being there for each other

The program made caregivers more aware about the relationship with their partner and the time they spent together. The program stimulated partners to think about the activities caregivers undertook with their partner. This made some caregivers decide they wanted to make changes, for example to spend more time with their partner:

*“We gained from the program that we have more attention for each other. I sometimes play games and go to bed late, and now I’m trying to reduce that. And this has been successful. [...]  So we go to bed at the same time. I still use that. And that is something you become more aware of during the program.” (R12)*

Paying more attention to the spousal relationship also meant that caregivers were more inclined to share their emotions and thoughts with their partner. The program helped to enhance the communication with their partner as it encouraged caregivers to discuss topics with their partner in the exercises. Due to these exercises, caregivers communicated about their emotions and difficult subjects with their partner in a way they had not done before:

*“[…] talking about the funeral, the preparations. That is something we don’t discuss and we didn’t discuss before. But because of the program, we have started talking about it.” (R1)*

Some couples jointly decided that they would start to talk about certain difficult topics such as life prolonging measures, when these would become relevant in their disease stage. Being on the same page with their partner with regard to these difficult topics made caregivers feel less stressed. Others felt the information in the program improved their understanding of their partner’s needs and emotions. This increased understanding helped to improve the communication between them and their partner:

*“I now recognize the reactions from my partner better and understand that she can experience different emotions and needs than I do. I now try to anticipate.” (R9)*

#### Feeling acknowledged

Caregivers were pleased to receive attention that was specifically intended for them: this made them feel heard and understood. It acknowledged that their role is important in the care process and reduced the feeling of being on their own.

*“It has to do with the fact that it was aimed at me. That is what I enjoyed about it so much. The fact that I did something that was completely focused on me, that felt very nice.” (R13)*

Caregivers felt the information matched their situation well. Some of the strategies aimed at dealing with difficult situations and emotions introduced in the program were recognised by them as strategies they already applied in their daily lives. The intervention, therefore, endorsed that they were doing well in terms of, for example, discussing difficult subjects or planning quality time together. This confirmation gave caregivers a positive feeling and made them feel more self-confident about their coping strategies:

*“I can remember that sometimes I would think: ‘hey, I’m doing alright.’ Cause there would be tips on how you could do things and they appealed to me and they supported me in a sense that I already did those things myself or already thought about that way.” (DR4)*

By participating in the support program, caregivers had to spend time on reading and completing exercises. Some caregivers rarely sat down and took time for themselves and they experienced this as a positive effect of the support program.

*“[…] that you had to take time for yourself. […] that you’re more or less forced to sit down calmly and to think about things for a bit. That worked for me.” (R5)*

## Discussion

Caregivers evaluated the support program positively. They indicated that the support program increased their awareness with regard to their own situation. Having a partner who received a diagnosis of ALS or PMA is experienced as a highly demanding and overwhelming situation [19]. Reflecting on their current situation and their role therein does not seem self-evident for caregivers who are in the constant flow of providing care. The program “forced” caregivers to reflect on and become more aware of their situation which was considered as a crucial function of the program by caregivers. Becoming aware of your own situation is one of the first stages that people go through during the process of change that takes place in psychological therapy [20]. In the stages that follow, people modify their behavior, experiences, and environment to overcome difficulties [20]. Becoming more aware of their own situation made caregivers in the current study realize they wanted or needed to do things differently in order to remain healthy.

Due to the program, caregivers perceived more control over their care situation, accepted negative emotions and thoughts, reported increased attention to their partner relationship and felt acknowledged. The program empowered caregivers to make choices according to their own needs which they perceived as a positive change. This is in line with the rationale of the demand and control model, in which increased perceived feelings of control act as a protective buffer against the impact of perceived demands on the wellbeing of the caregiver [3]. Furthermore, previous research has shown that accepting negative emotions and thoughts has positive effects on the wellbeing of individuals [21]. Our study showed that this is also beneficial for the partner relationship; caregivers became more aware of their emotions and thoughts and talked about these topics with their spouse. It has been demonstrated that sharing emotions and thoughts and communicating about disease-related topics with your partner leads to increased feelings of intimacy [22–24], while hiding worries and dismissing negative emotions are associated with more perceived distress [25, 26].

Caregivers are often inclined to neglect their own needs and wellbeing [6]. Our study revealed that caregivers appreciated a support program specifically aimed at them because the attention from professionals and the social network is mostly focused on the patient [27]. This indicates that there is an unmet underlying need for support. Providing support online may lower the threshold to accept support for these caregivers.

Caregivers in this study valued the online support because they were able to enter the program at their preferred time and place, and could work on the program at their own pace. These benefits were also reported in other studies for caregivers [28–30]. Since these caregivers are often occupied with care tasks which makes it complicated to receive face-to-face support, using online support seems to be a suitable way to provide support and to reach out to the caregivers who are in need of care [31].

Overall, the different components of the support program were appreciated by the caregivers, but they expressed mixed opinions regarding the components mindfulness and peer support. Although mindfulness based interventions has been shown to decrease feelings of depression and caregiver burden in caregivers [32, 33], most caregivers in our study did not perceive the mindfulness exercises as helpful. Yet, it is not uncommon for participants to report unpleasant reactions, such as agitation, discomfort, or confusion during mindfulness interventions [34]. These reactions are viewed as part of the psychological process, since mindful attention to one’s reactions is thought to help participants explore and understand these reactions [34]. Providing more information and support with regard to this process might be needed. Another important remark here is that caregivers, who perceived the mindfulness exercises as helpful, were those with prior experience with mindfulness of meditation.

The other component that received mixed evaluations was the option of peer contact. The majority of the caregivers mentioned that they were not in need of peer contact or they thought talking to others in a more advanced stage would be too confronting. These results are in line with the results in previous studies, which concluded that the fear of negative prospects can prevent participants from seeking peer contact [35, 36]. Although peer support can have advantages, having contact with others who are coping well can provide hope and generate information which positively impacts upon one’s own problem solving skills [36, 37]. But it might not be suitable for everyone. Providing mindfulness and peer contact as optional parts of the support program is recommended for future implementation.

### Clinical implications

Caregivers stressed that the timing of interventions should match their needs for them to perceive the intervention as helpful. However, the right timing for a support intervention might differ for each individual caregiver. In addition, the needs of caregivers change during the disease course [38] and they seem to be reluctant to seek support for themselves [39]. Acknowledging the important role of these caregivers in the care of patients at an early stage and underlining their risk of psychological distress by care professionals, are crucial to lowering the boundaries for caregivers to accept the support offered. Receiving interventions early in the disease trajectory may better prepare caregivers for what is yet to come and provide them with tools in order to prevent caregiver distress in the future. For caregivers of patients in a more advanced stage of the disease, receiving support is difficult, as they might not have enough time to spent on support or they may no longer be able to benefit from it due to the progressed disease stage. Therefore, it is recommended that information be provided about the support program, as well as other supportive interventions by the multidisciplinary ALS care teams, in an early phase of the disease and repeatedly thereafter [31].

Another way of making care more accessible is by providing personalized support where caregivers can choose options. In case of the support program, following modules in a self-chosen order and time may increase the perceived acceptability and value of the intervention. This is in line with the current focus in the field of caregiver interventions; targeting interventions to specific caregiving groups and subsequently tailoring those to individual caregiver’s needs [40]. Offering tailored interventions according to the needs of the recipient reduces negative effects of interventions, decreases waste of time and effort of both recipient and professional, and may increase the compliance with the intervention [41].

### Strengths

This study was nested within a RCT and may provide information that enhances the understanding of the results of the trial and the implementation once the effectiveness has been established. To strengthen the trustworthiness of the study, data were independently analysed by two researchers and supported by a qualitative research expert during the process of analysis. Memo writing, the use of the checklist of Braun & Clark and the SRQR checklist further enhanced the trustworthiness [17, 18]. Furthermore, the interviewer was unknown to the participants prior to the interview and was not part of the trial, which might have positively affected the representativeness of the results.

### Limitations

A few limitations need to be considered. First, interviews were conducted after completing the intervention and this retrograde reflection might not have revealed all experiences with the intervention. Second, there was a delay between completing the intervention and the interview, which might have affected caregivers’ memory to recall the details of the intervention. Therefore, a short summary of all modules of the intervention was provided a few days before the interview to help retrieve caregivers’ memory. A third limitation is that the interviews were conducted by telephone for logistic reasons. Face-to-face interviews could have encouraged caregivers to further elaborate on their answers which may have enriched the data [42]. However, telephonic interviews may allow respondents to disclose sensitive information more freely [43].

## Conclusions

Partners of patients with ALS and PMA overall appreciated the blended psychosocial support program based on ACT but they expressed mixed feelings about the mindfulness and peer support components. The program increased their awareness with regard to their own situation; increased their perceived control over the care situation; helped to accept negative emotions and thoughts; increased their attention for their partner relationship and acknowledged them. Our program should be considered as a complementary approach to multidisciplinary ALS care in which the important role of these caregivers and their risk of distress and burden are acknowledged. Offering interventions by the ALS care team early in the disease course and repeatedly thereafter is preferable, as care needs change over time. Providing information about the content of the program and subsequently tailoring the program to the specific needs of the caregivers (i.e. caregivers choose which module at what time and which pace) may increase the perceived benefits and compliance with the intervention.

## Additional files

Additional file 1: A description of the content of the psychosocial support program (DOCX 25 kb)

Additional file 2: The guidelines that were used for the interviews with caregivers. (DOCX 27 kb)

Additional file 3: A checklist for the thematic analysis of qualitative data. (DOCX 33 kb)

Additional file 4: A standard checklist for reporting qualitative research. (DOCX 35 kb)

## Data Availability

The datasets generated and analyzed during the current study are available from the author upon reasonable request.

## Abbreviations

ACT: Acceptance and Commitment Therapy
ALS: Amyotrophic Lateral Sclerosis
ALS-FRS-R: Amyotrophic Lateral Sclerosis Functional Rating Scale-Revised
ALS-FTD-Q: Amyotrophic Lateral Sclerosis-Fronto Temporal Dementia-Questionnaire
DR: Drop-out Respondent
PMA: Progressive Muscular Atrophy
R: Respondent
RCT: Randomized Controlled Trial
SRQR: Standards for Reporting Qualitative Research; yrs.:years
