# Supplementary material for: User perspectives on a psychosocial blended support program for partners of patients with amyotrophic lateral sclerosis and progressive muscular atrophy: a qualitative study
Source: BMC Psychol. 2019 Jun 15;7:35. doi: 10.1186/s40359-019-0308-x (PMC6570885; doi:10.1186/s40359-019-0308-x)
Supplement: Supplementary file 1 — A description of the content of the psychosocial support program (DOCX 25 kb) [file 40359_2019_308_MOESM1_ESM.docx]

| Additional file 1. Content intervention | | |  | |
| --- | --- | --- | --- | --- |
| Part of intervention | Topics | Goals | | Key components |
| Face-to-face session | - The care situation - Wellbeing caregiver - Information about support program - Log in online modules | To receive information about the care situation and establish a relationship between the psychologist and the caregiver. To inform about caregiver burden and start online modules. | | - Psychoeducation |
| Online module 1  Coping with your emotions and thoughts | - Dealing with and expressing emotions - Recognizing thoughts | To recognize emotions and encourage caregivers to allow, express and share emotions that can arise. To recognize dysfunctional thoughts and rumination. Change the way the caregiver relates to thoughts/ to create distance from thoughts. | | - Acceptance - Cognitive defusion - Mindfulness |
| Online module 2  The art of communication | - Communication style - Communicating about sensitive topics - Communication about providing care | To improve the overall communication and to communicate with the patient about sensitive topics and providing care in the future. | | - Communicating about what really matters - Mindfulness |
| Online module 3  Your resilience plan | - Dealing with continuous stress - Moments of relaxation - Using your sources | To make a resilience plan that may allow caregivers to maintain health during this stressful period by taking care of themselves. | | - Acceptance - Mindfulness |
| Online module 4  What is really important | - Values in relationship - Values in life | To identify the values of the caregiver in different areas of life and to plan actions to meet these values. | | - Values - Commited action - Mindfulness |
| Online module 5  Moments of joy | - Positivity during difficult times - Celebrate the relationship | To seek, enjoy and cherish the positive moments in the relationship and in life. | | - Committed action - Mindfulness |
| Online module 6  A good last period | - Life story of the patient - Communication in this last phase - Beautiful memories - Being grateful | To create a beautiful last period with the loved ones and to make memories with the patient for the future. | | - Acceptance - Communicating about what really matters - Committed action - Mindfulness |
| Telephone call | - Any questions - Finish the support program | To offer support with regard to any issues and close the support program. | |  |
