# Supplementary material for: User perspectives on a psychosocial blended support program for partners of patients with amyotrophic lateral sclerosis and progressive muscular atrophy: a qualitative study
Source: BMC Psychol. 2019 Jun 15;7:35. doi: 10.1186/s40359-019-0308-x (PMC6570885; doi:10.1186/s40359-019-0308-x)
Supplement: Supplementary file 2 — The guidelines that were used for the interviews with caregivers. (DOCX 27 kb) [file 40359_2019_308_MOESM2_ESM.docx]

Additional file 2. Interview guidelines

Topics interviews with completers

- *Experiences with the program in general*
- *Motivation to start with the program*
- *Perceived benefits of the program*
- *Perceived disadvantages of the program*
- *Match of the program with the needs of the caregiver*
- *Timing of the program*
- *Structure of the program*
- *Receiving online support*
- *Experiences with the different components of the program:*
  - *Home visit*
  - *Psychoeducation*
  - *Psychological exercises*
  - *Mindfulness*
  - *Information, tips and references*
  - *Contact with peers*
  - *Feedback from the counselor*
  - *Contact with the counselor*

Topics interviews with drop out

- *Reasons for dropping out*
- *Expectations of the program before the start*
- *Perceived benefits of the program*
- *Perceived disadvantages of the program*
- *Elements that could have prevented dropping out*
