# Supplementary material for: User perspectives on a psychosocial blended support program for partners of patients with amyotrophic lateral sclerosis and progressive muscular atrophy: a qualitative study
Source: BMC Psychol. 2019 Jun 15;7:35. doi: 10.1186/s40359-019-0308-x (PMC6570885; doi:10.1186/s40359-019-0308-x)
Supplement: Supplementary file 3 — A checklist for the thematic analysis of qualitative data. (DOCX 33 kb) [file 40359_2019_308_MOESM3_ESM.docx]

Additional file 3**.** Checklist of Criteria for Good Thematic Analysis: 15-point checklist

| **Process** | **Criteria** | **Reported in chapter** |
| --- | --- | --- |
| Transcription | 1. The data have been transcribed with an appropriate level of detail, and the transcripts have been checked against the tapes for ‘accuracy’. | YES |
| Coding | 2. Each data item has been given equal attention in the coding process. | YES |
|  | 3. Themes have not been generated from a few vivid examples (an anecdotal approach), but instead the coding process has been thorough, inclusive and comprehensive. | YES |
|  | 4. All relevant extracts for each theme have been collated. | YES |
|  | 5. Themes have been checked against each other and against the original data set. | YES |
|  | 6. Themes are internally coherent, consistent, and distinctive. | YES |
| Analysis | 7. Data have been analysed – interpreted, made sense of - rather than just paraphrased or described. | YES |
|  | 8. Analysis and data match each other – the extracts illustrate the analytical claims. | YES |
|  | 9. Analysis tells a convincing and well-organised story about the data and topic. | YES |
|  | 10. A good balance between analytical narrative and illustrative extracts is provided. | YES |
| Overall | 11. Enough time has been allocated to complete all phases of the analysis adequately, without rushing a phase or giving it a once-over-lightly. | YES |
| Written  report | 12. The assumptions about, and specific approach to, thematic analysis are clearly explicated. | YES |
|  | 13. There is a good fit between what you claim you do, and what you show you have done – i.e., described method and reported analysis are consistent. | YES |
|  | 14. The language and concepts used in the report are consistent with the epistemological position of the analysis. | YES |
|  | 15. The researcher is positioned as *active* in the research process; themes do not just ‘emerge’. | YES |

Adapted from: Braun, V., & Clarke, V. Using thematic analysis in psychology. *Qualitative research in psychology.2006; 3*(2), 77-101.
